# The Effects of a Blood–Brain Barrier Penetrating Erythropoietin in a Mouse Model of Tauopathy

**DOI:** 10.3390/ph16040558

**Published:** 2023-04-07

**Authors:** Joshua Yang, Weijun Ou, Nataraj Jagadeesan, Juste Simanauskaite, Jiahong Sun, Demi Castellanos, David H. Cribbs, Rachita K. Sumbria

**Affiliations:** 1 Henry E. Riggs School of Applied Life Sciences, Keck Graduate Institute, 535 Watson Dr, Claremont, CA 91711, USA; 2 Department of Biomedical and Pharmaceutical Sciences, School of Pharmacy, Chapman University, Irvine, CA 92618, USA; 3 Department of Neuroscience, Pomona College, Claremont, CA 91711, USA; 4 Institute for Memory Impairments and Neurological Disorders, University of California, Irvine, CA 92697, USA; 5 Department of Neurology, University of California, Irvine, CA 92868, USA

**Keywords:** Alzheimer’s disease, blood–brain barrier, erythropoietin, microglia, molecular Trojan horse, monoclonal antibody, phospho-tau, transferrin receptor

## Abstract

Erythropoietin (EPO), a hematopoietic neurotrophin, is a potential therapeutic for Alzheimer’s disease (AD) but has limited blood–brain barrier (BBB) permeability. EPO fused to a chimeric transferrin receptor monoclonal antibody (cTfRMAb) enters the brain via TfR-mediated transcytosis across the BBB. We previously showed that cTfRMAb-EPO is protective in a mouse model of amyloidosis, but its effects on tauopathy are not known. Given that amyloid and tau pathology are characteristics of AD, the effects of cTfRMAb-EPO were studied in a tauopathy mouse model (PS19). Six-month-old PS19 mice were injected intraperitoneally with either saline (PS19-Saline; *n* = 9) or cTfRMAb-EPO (PS19-cTfRMAb-EPO, 10 mg/kg; *n* = 10); every two or three days on alternate weeks for 8 weeks. Age-matched, saline-treated, wildtype littermates (WT-Saline; *n* = 12) were injected using the same protocol. After 8 weeks, locomotion, hyperactivity, and anxiety were assessed via the open-field test, and brains were harvested and sectioned. Cerebral cortex, hippocampus, amygdala, and entorhinal cortex sections were analyzed for phospho-tau (AT8) and microgliosis (Iba1). Hippocampal cellular density (H&E) was also assessed. PS19-Saline mice were hyperactive and less anxious compared to WT-Saline mice, and these behavioral phenotypes were significantly reduced in the PS19-cTfRMAb-EPO mice compared to the PS19-Saline mice. cTfRMAb-EPO significantly reduced AT8 load by ≥50% in all of the brain regions analyzed and microgliosis in the entorhinal cortex and amygdala compared to the PS19-Saline mice. Hippocampal pyramidal and granule cell layer density did not differ significantly between the PS19-cTfRMAb-EPO and PS19-Saline mice. This proof-of-concept study demonstrates the therapeutic effects of the BBB-penetrating cTfRMAb-EPO in PS19 mice.

## 1. Introduction

Alzheimer’s disease (AD) is a progressive neurodegenerative disease that is characterized by the deposition of fibrous senile amyloid-beta (Aβ) plaques and intraneuronal aggregates of hyperphosphorylated tau known as neurofibrillary tangles (NFTs) [1]. Erythropoietin (EPO) is a 30.4 kDa hematopoietic growth factor that has neuroprotective effects such as reducing inflammation, oxidative stress, and neuronal loss while promoting neurogenesis and angiogenesis [2,3,4]. Therefore, EPO provides neuroprotective and neuroregenerative effects that are likely to provide a disease-modifying strategy for AD. However, due to its large molecular weight and polarity, EPO cannot readily enter the brain parenchyma due to the blood–brain barrier (BBB) [5,6,7], and it therefore requires high doses that can increase adverse hematopoiesis [8].

Transferrin receptor 1 (TfR), which is highly enriched at the BBB [9], is a receptor-mediated transcytosis (RMT) system that regulates intracellular iron and iron transport across the BBB through its binding to the iron-binding protein transferrin [10]. Accordingly, an antibody directed against the TfR that binds to an epitope separate from the transferrin binding site can be used as a molecular Trojan horse (MTH) to deliver biologics into the brain parenchyma via the transvascular route using this RMT approach [11]. TfR-targeted therapies have also been tested for cancers, given the overexpression of TfR in cancer cells [12]. To study the effects of EPO in AD mouse models via a non-invasive transvascular route of administration, an MTH-EPO fusion protein was engineered using a rat/mouse chimeric antibody directed against the mouse TfR (cTfRMAb) and EPO [13]. The cTfRMAb-EPO fusion protein binds to the TfR and EPO receptors with high affinity; the former enables brain delivery of EPO via interaction with the BBB’s TfR, and the latter results in neuroprotective effects from EPO via the EPO receptor in the brain [13,14]. Further, cTfRMAb-EPO also binds to the peripheral TfR and enhances its clearance from the systemic circulation compared to EPO [13], and this is expected to reduce the hematopoietic adverse effects associated with high circulating concentrations of EPO [15]. cTfRMAb-EPO is therefore a BBB-penetrable EPO which is expected to have negligible adverse hematopoietic effects.

Our previous work shows that the cTfRMAb-EPO reduces Aβ plaques and insoluble Aβ (1–42) levels in the APP/PSEN1 transgenic mouse model of amyloidosis [15,16]. However, the effect of cTfRMAb-EPO on hyperphosphorylated tau, the primary constituent of NFTs, which are a characteristic neuropathologic marker of tauopathies including AD [17], has not been studied. Although there is a growing body of literature reporting the protective effects of EPO on amyloid pathology [18], studies reporting the effect of EPO on Aβ-independent tau pathology are limited. In this respect, the use of a low molecular weight EPO-derived peptide that penetrates the BBB was reported to mitigate neurological deficits and neuropathological changes in female PS19 mice [19]. The PS19 mice express the P301S mutant human tau, resulting in hyperphosphorylated tau and NFT-like inclusions with age [20], microgliosis and astrocytosis [21], and age-dependent brain atrophy and neuronal loss in the hippocampus, neocortex, and entorhinal cortex [21] in the absence of Aβ pathology. 

Given the potential protective effects of EPO on tauopathy, the current study aimed to investigate the effect of the BBB-penetrating EPO (cTfRMAb-EPO) on tau pathology. The effects of cTfRMAb-EPO on phosphorylated tau, microgliosis, hippocampal neuronal loss, and behavior abnormalities were studied in the PS19 mice (Supplemental Figure S1A). 

## 2. Results

The weights of the mice at the beginning of the study were 35 ± 1 g, 31 ± 2 g, and 32 ± 1 for the WT, PS19-Saline, and PS19-cTfRMAb-EPO mice, respectively, and after 8 weeks, they were 35 ± 1 g, 31 ± 2 g, and 30 ± 1 g, respectively (Supplemental Figure S1B). There was no significant difference in mouse body weights between any experimental group compared to the PS19-Saline mice throughout the study and within each experimental group at baseline and following 8 weeks of treatment. No mice were lost during the study due to premature death or adverse events.

### 2.1. BBB-Penetrating EPO Reversed the Altered Anxiety and Hyperactive Phenotype of the PS19 Mice

**Figure 1:**
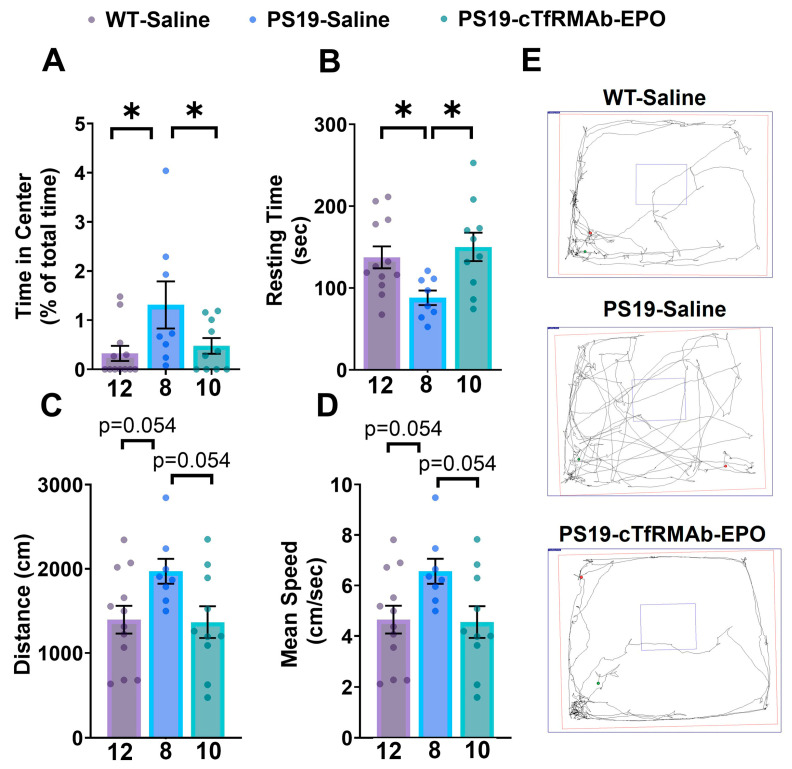
**Effect of cTfRMAb-EPO on anxiety and hyperactivity in the PS19 mice.** (**A**) The saline-treated PS19 mice spent more time in the center, (**B**) had reduced resting time, and (**C**) trended towards a higher distance traveled and (**D**) overall mean speed compared to the saline-treated WT mice during open-field testing. Chronic cTfRMAb-EPO treatment prevented these altered behaviors in the PS19 mice. (**E**) Representative trajectory maps showing the movement of the WT and PS19 mice. One outlier was excluded from the PS19-Saline group. One-way ANOVA with Holm–Sidak’s post hoc test was used to compare to the PS19-Saline mice. Data are shown as mean ± SEM of *n* = 8–12 per group (shown below each column). * *p* < 0.05 compared to PS19-saline.

The saline-treated PS19 mice spent significantly more time in the center of the open-field apparatus compared to the saline-treated WT mice (*p* < 0.05). cTfRMAb-EPO treatment significantly reduced the time spent in the center by the PS19 mice to values comparable to the WT mice’s values (*p* < 0.05; Figure 1A,E). 

Concerning hyperactivity and locomotion, the saline-treated PS19 mice had significantly reduced resting time compared to the saline-treated WT mice (*p* < 0.05), and cTfRMAb-EPO treatment increased the resting time of the PS19 mice back to the WT mice’s values (*p* < 0.05) (Figure 1B,E). A similar trend was observed in the overall mean speed and distance traveled by the mice, wherein the saline-treated PS19 mice trended towards a greater distance travelled and a higher mean speed compared to the saline-treated WT mice (*p* = 0.054), though these values did not reach statistical significance. There was a trend towards a reduction in the increased distance and mean speed with cTfRMAb-EPO treatment of PS19 mice (*p* = 0.054) (Figure 1C–E). 

### 2.2. BBB-Penetrating EPO Decreased Phosphorylated Tau (pTau) at Ser202 and Thr205 by Half in the PS19 Mice

**Figure 2:**
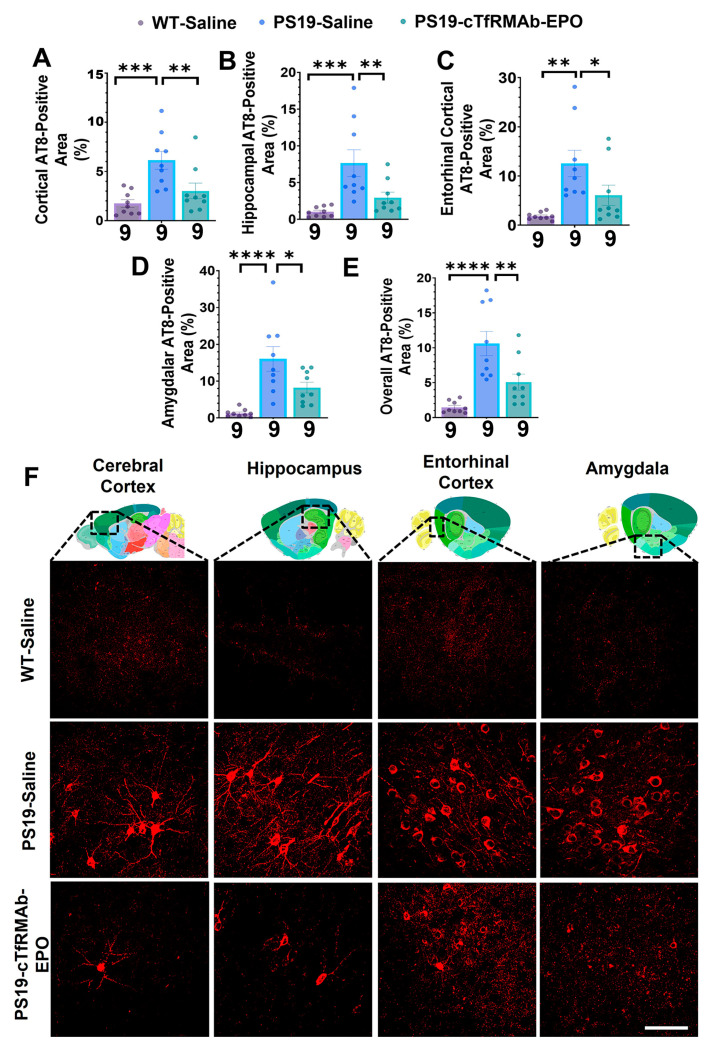
**Effect of cTfRMAb-EPO on the pTau-positive area in the PS19 mice with AT8 immunostaining.** The AT8-positive area was significantly lower in the cTfRMAb-EPO-treated PS19 mice compared to the saline-treated PS19 mice in the (**A**) cortex, (**B**) hippocampus, (**C**) entorhinal cortex, and (**D**) amygdala. (**E**) The overall AT8-positive area, which is the average of all brain regions, was also significantly reduced with cTfRMAb-EPO. (**F**) Representative images of AT8-positive pTau with thumbnail brain section images adapted from the Allen Institute that show the cerebral cortex, hippocampus, entorhinal cortex, and amygdala. Data are presented as mean ± SEM of *n* = 9 per treatment group (shown below each column). One outlier was excluded from the WT-Saline and PS19-cTfRMAb-EPO groups. One-way ANOVA with Holm–Sidak’s post hoc test was used to compare to the PS19-Saline group. * *p* < 0.05, ** *p* < 0.01, *** *p* < 0.001, **** *p* < 0.0001. Scale bar = 100 μm.

The AT8-positive area was significantly higher (72–92% higher, *p* < 0.01) in the saline-treated PS19 mice compared to the saline-treated WT mice in all brain regions (Figure 2A–F). The AT8-positive area in the cTfRMAb-EPO-treated PS19 mice was significantly reduced in the cerebral cortex (51% reduction; *p* < 0.01; Figure 2A,F), hippocampus (62% reduction, *p* < 0.01; Figure 2B,F), entorhinal cortex (52% reduction, *p* < 0.05; Figure 2C,F), amygdala (49% reduction, *p* < 0.05; Figure 2D,F), and overall (average of all regions) (52% reduction; *p* < 0.01; Figure 2E,F) compared to the saline-treated PS19 mice. We also observed a trend (without statistical significance) towards a decrease in total plasma tau after 8 weeks of treatment with cTfRMAb-EPO compared to the saline-treated PS19 mice (*p* = 0.077; Supplemental Figure S2).

### 2.3. BBB-Penetrating EPO Reduced Microgliosis in the Entorhinal Cortex and Amygdala of the PS19 Mice

**Figure 3:**
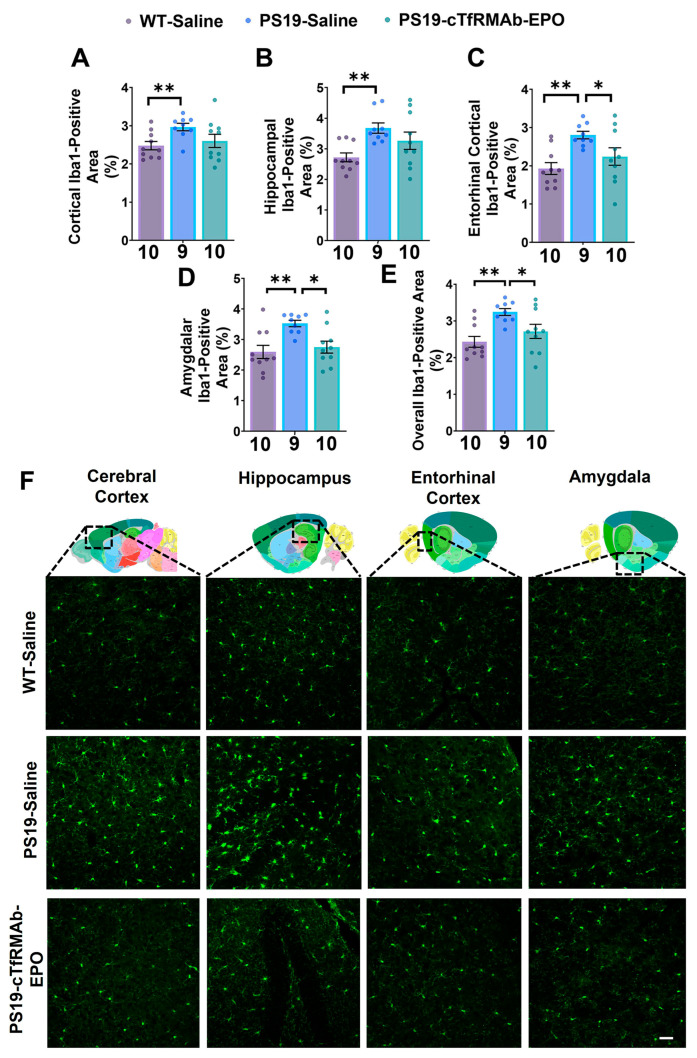
**Effect of cTfRMAb-EPO on microgliosis in the PS19 mice with Iba1 immunostaining.** (**A**–**F**) The Iba1-positive area in different brain regions. The Iba1-positive area was significantly lower in the cTfRMAb-EPO-treated PS19 mice compared to the saline-treated PS19 mice in the entorhinal (**C**) cortex and (**D**) amygdala. (**E**) The overall Iba1-positive area, which is the average of all the brain regions, was also significantly reduced with cTfRMAb-EPO. (**F**) Representative images of Iba1-positive microglia with thumbnail brain section images adapted from the Allen Institute that show the cerebral cortex, hippocampus, entorhinal cortex, and amygdala. Data are presented as mean ± SEM of *n* = 9–10 per treatment group (shown below each column). No outliers were detected. One-way ANOVA with Holm–Sidak’s post hoc test was used to compare to the PS19-Saline group. * *p* < 0.05, ** *p* < 0.01. Scale bar = 100 μm.

The Iba1-positive area was significantly higher (17–31% higher, *p* < 0.01) in the saline-treated PS19 mice compared to the saline-treated WT mice (Figure 3A–F). cTfRMAb-EPO treatment lowered the Iba1-positive area in the entorhinal cortex (20% lower; *p* < 0.05; Figure 3C,F) and amygdala (22% lower, *p* < 0.05; Figure 3D,F) compared to the saline-treated PS19 mice. The overall (average of all the brain regions) Iba1-positive area was also significantly lower (16% lower, *p* < 0.05) in the cTfRMAb-EPO-treated PS19 mice compared to the saline-treated PS19 mice (Figure 3E,F). 

### 2.4. BBB-Penetrating EPO Did Not Alter Hippocampal Neuronal Density in the PS19 Mice

**Figure 4:**
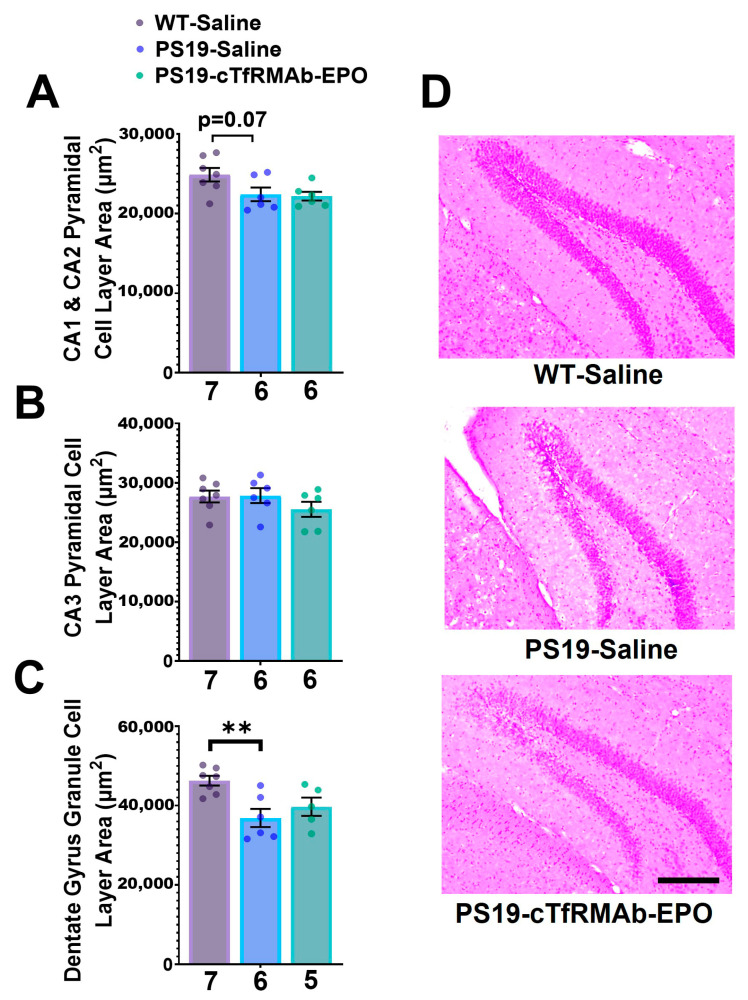
**Effect of cTfRMAb-EPO on hippocampal pyramidal and granule cell layers with H&E staining.** (**A**) Pyramidal cell layer area in the CA1 and CA2 and (**B**) CA3, and (**C**) granule cell layer area in the dentate gyrus (DG). (**C**) The granule cell layer area was significantly higher in the saline-treated WT mice compared to the saline-treated PS19 mice in the DG. No significant effect of cTfRMAb-EPO treatment was observed on the (**A**,**B**) pyramidal or (**C**) granule cell layer areas compared to the PS19 saline-treated mice. (**D**) Representative images of the H&E-stained hippocampal granule cell layer of the DG region. Data are presented as mean ± SEM of *n* = 5–7 per treatment group (shown below each column). One outlier was excluded from the PS19-cTfRMAb-EPO group in panel C. One-way ANOVA with Holm–Sidak’s post hoc test was used to compare to the PS19-Saline mice. ** *p* < 0.01. Scale bar = 200 μm.

Hippocampal neuronal health was determined by quantifying the area occupied by the pyramidal cell layer in the CA1–3 regions and the granule cell layer in the dentate gyrus (DG) of the hippocampus. There was a trend (without statistical significance) towards reduction in the CA1–CA2 pyramidal cell layer area in the PS19-Saline mice compared to the saline-treated WT mice (*p* = 0.07, Figure 4A). There was no significant difference in the pyramidal cell layer area of the CA3 region between the groups (Figure 4B). The hippocampal granule cell layer area in the PS19-Saline mice was significantly lower in the DG (20% lower; *p* < 0.01) compared to the saline-treated WT mice (Figure 4C,D). The administration of cTfRMAb-EPO did not prevent reduction in the pyramidal or granule cell layer areas in the PS19 mice (Figure 4).

## 3. Discussion

cTfRMAb-EPO is a bifunctional, molecule wherein the cTfRMAb domain ferries the EPO into the brain by binding to the BBB’s TfR [13]. TfR-mediated transcytosis of cTfRMAb-EPO into the brain was shown using the capillary depletion method, which separates the brain parenchyma from the brain vasculature [13]. The cTfRMAb-EPO used in the current study has a single mutation in the Fc N-linked glycosylation site at position 292 where the amino acid asparagine (Asn) is substituted with glycine (Gly) (N292G mutation), making the fusion protein aglycosylated, to reduce the Fc-effector function associated adverse effects [14]. The N292G mutant, however, has a lower plasma exposure and faster plasma clearance than cTfRMAb-EPO without the N292G mutation [14]. Therefore, higher doses of cTfRMAb-EPO with the N292G mutation are needed to produce therapeutic effects comparable to those of cTfRMAb-EPO without the N292G mutation [14]. Our prior work shows that intraperitoneal (IP) doses between 9–20 mg/kg of the N292G mutant cTfRMAb-EPO will result in plasma concentrations similar to those obtained with the therapeutic 3 mg/kg dose of cTfRMAb-EPO without the N292G mutation [22]. Therefore, in the current proof-of-concept study, we used the 10 mg/kg IP dose of the cTfRMAb-EPO with the N292G mutation. Future work will use lower doses of cTfRMAb-EPO without the N292G mutation. 

The brain uptake of a cTfRMAb-based fusion protein without the N292G mutation following a 3 mg/kg dose (comparable to a 10 mg/kg dose of cTfRMAb-EPO with the N292G mutation used herein) is between 1.25 µg/kg and 0.7 µg/kg in a mouse at 6 h and 24 h after IP injection, respectively [23]. Based on this, the expected brain concentration of EPO is between 140 to 250 ng/g brain (20% of cTfRMAb-EPO). EPO is a potent neurotrophin, and such concentrations of EPO in the brain parenchyma are expected to exert neuroprotective effects. Notably, concentrations as low as 0.2 ng/g brain of EPO are reported to be effective in preventing neuronal apoptosis [24]. The protective effects of EPO are primarily produced by binding of a single EPO molecule to a dimer of the cytokine-type-1 transmembrane receptor. Within the mouse brain, EPO receptor expression was shown not only in the neurons, but also in the microglia, astrocytes, and oligodendrocytes [25]. Therefore, the protective effects of EPO can be mediated by acting on different cell types in the CNS [26]. In addition to enabling blood to brain delivery of the EPO, once in the brain, the cTfRMAb domain of the fusion protein can also facilitate the uptake of the fusion protein into other TfR-expressing cells, including the neurons and glial cells [27,28,29,30], to exert protective effects in these cells.

PS19 mice exhibit behavioral abnormalities such as hyperactive locomotor activity and reduced anxiety caused by mutant tau overexpression [31,32]. We used the open-field testing paradigm to gain insights into the locomotor and anxiety-related behaviors in the PS19 mice. Rodents without behavioral modifications spend significantly more time exploring the periphery of the open-field maze, and mice that spend more time in the center of the maze illustrate less anxiety-like behavior [33]. The saline-treated PS19 mice spent significantly more time in the center in the current study, demonstrating reduced anxiety-like behavior that is consistent with the PS19 mouse model of tauopathy (Figure 1A) [34]. The saline-treated PS19 mice also displayed increased locomotion hyperactivity, which is also consistent with the PS19 mouse model of tauopathy (Figure 1) [34,35]. These mice had significantly lower resting times compared to WT mice (Figure 1B). Though we cannot rule out the impact of hyperactivity (disinhibited behavior) on time spent in the center of the open-field apparatus, PS19 mice consistently show reduced anxiety-like behavior in other testing paradigms. For example, the elevated plus maze is widely used to assess anxiety-like behavior in mice [36], and PS19 mice spend more time in open arms than closed arms, which is indicative of the reduced anxiety-like behavior of these mice [34,37]. Chronic dosing of cTfRMAb-EPO both reduced locomotion hyperactivity and altered anxiety-like behavior in the PS19 mice, which suggests that administering the BBB-penetrating EPO analog had a positive effect in this mouse model of tauopathy (Figure 1). In addition to alterations in anxiety and locomotion, PS19 mice may show age-dependent cognitive deficits. Several studies report no or minor hippocampal-dependent cognitive deficits at 9 months of age in PS19 mice [35,38,39] and some studies report significant hippocampal-dependent cognitive deficits in PS19 mice [34]. Our previous work showed that locomotion hyperactivity was a significant correlate of AT8 immunoreactivity [40], and therefore, we focused on this measure in the current study.

The association between AT8 load and hyperactivity in PS19 mice [40] was further corroborated in the current study. Chronic treatment with the cTfRMAb-EPO fusion protein markedly reduced the AT8-positive pTau immunoreactive area in the PS19 mice compared to the saline-treated PS19 mice in all of the analyzed brain regions (Figure 2). This is consistent with a recent study that shows that an EPO-derived peptide can reduce AT8-positive pTau in 10-month-old PS19 mice [19]. Although the BBB-penetrating EPO did not completely reduce the AT8-positive area to the WT levels in our hands, the AT8-positive area was reduced by 50% or more compared to the saline-treated PS19 mice (Figure 2). We also measured the levels of total tau in the plasma, which is a correlate of CNS pTau [41], and our previous work shows that total plasma tau levels share a positive correlation with brain AT8 immunofluorescence positive area [40]. In the current study, we found a trend (without statistical significance) towards a reduction in plasma total tau in the cTfRMAb-EPO-treated PS19 mice compared to the saline-treated PS19 mice, paralleling the reduction in brain AT8-positive area with cTfRMAb-EPO (Supplemental Figure S2).

Although EPO was shown to have neuroprotective effects by reducing pTau and oxidative stress [18,42], the exact mechanisms underlying the reduction in Aβ-independent pTau are understudied. One potential mechanism by which EPO reduces pTau is by modulating microgliosis. Microglia are involved in maintaining brain homeostasis by surveilling and clearing any pathological proteins and debris from the brain’s extracellular space [43]. However, in AD, abnormal and continuous activation of microglia can lead to impairments in tau phagocytosis and a pro-inflammatory state [43,44]. This pro-inflammatory state can lead to the hyperphosphorylation of tau [45]. Synaptic loss and microglial activation precede the emergence of NFTs in PS19 mice [21], and misfolded protein tau aggregation and microglial activation correlate with clinical AD and act as key determinants in AD progression [46,47,48]. Further, microglial activation is a significant correlate of pTau both in human tauopathy [49,50] and in PS19 mice [21,45,51].

We found a significant increase in Iba1 immunoreactivity in the brains of the PS19 mice and a modest but significant reduction in Iba1 immunoreactivity with chronic cTfRMAb-EPO treatment (Figure 3). These results are consistent with the strong significant positive correlation between the overall AT8-positive area and the overall Iba1-positive area in the 8-month-old PS19 mice [40]. Though we cannot determine if the effects of cTfRMAb-EPO are due to a direct effect on tau phosphorylation and/or microgliosis, these findings suggest significant cross-talk between pTau and microgliosis, and this is consistent with the reduction in phosphorylated tau aggregation and microglial activation observed with the 19’mer EPO-derived cyclic peptide in PS19 mice [19]. 

In the current study, significant neuronal loss was observed only in the granule cell layer of the DG region of the hippocampus (Figure 4C), with a trend towards a reduction in the pyramidal cell layer in the CA1 and CA2 regions of the hippocampus. Hippocampal neuronal loss in H&E-stained sections was reported in 12-month-old PS19 mice [21], and the modest hippocampal neuronal loss in the current study is possibly caused by the younger age of the mice at the time of sacrifice (8-months-old). Tau-induced neurodegeneration may not have progressed far enough to display significant losses in the other hippocampal regions at this age. These results are consistent with the progression of neuronal loss in PS19 mice [21]. Although 6-month-old PS19 mice do not experience significant neuronal loss, these mice have widespread tau-positive neuronal staining and microgliosis [21], implying that neuronal loss follows tau phosphorylation and microgliosis in this mouse model. Chronic treatment with cTfRMAb-EPO did not prevent the small but significant hippocampal neuronal loss in the PS19 mice (Figure 4), despite its therapeutic effects in reducing hippocampal pTau (Figure 2) and despite our previous work showing an improvement in neuronal health with this BBB-penetrating EPO in an APP/PSEN1 mouse model of amyloidosis [15]. This observation was also contrary to the protective effect of the EPO-derived peptide on hippocampal neuronal loss in 10-month-old PS19 mice that display more robust hippocampal neuronal loss. The absence of a therapeutic effect of the BBB-penetrating EPO on hippocampal neuronal health may be attributed to limited hippocampal neuronal loss observed in the 8-month-old mice, and future studies using older PS19 mice that are expected to show more widespread neuronal loss will be needed to determine the effect of the BBB-penetrating EPO on pTau lesion-induced neuronal loss. 

The main side effect associated with chronic EPO dosing is adverse hematopoiesis [8]. The doses of EPO used in experimental AD studies range between 500–5000 IU/kg, with 5000 IU/kg (50 µg/kg) being the most widely used dose, given the limited BBB penetration of EPO [5,18]. These EPO doses result in an improvement in cognitive deficits and reduction in cerebral Aβ; however, they are associated with a significant increase in hematocrit after 4 to 12 weeks of dosing [52,53]. For comparison, at doses between 3 and 20 mg/kg of cTfRMAb-EPO with the N292G mutation, no changes in red blood cell count or hematocrit were observed after a single injection [22]. However, cTfRMAb-EPO with the N292G mutation was associated with a significant reduction in reticulocytes [22], immature red blood cells with high TfR expression to meet the iron demands during red blood cell maturation within the bone marrow [54]. Reticulocyte suppression has been observed with cTfRMAb-based therapies but is found to be short-lived and reversible [55]. Accordingly, chronic 6- to 8-week dosing with cTfRMAb-EPO without the N292G mutation [15] or other cTfRMAb-based therapies [56] does not result in reticulocyte suppression in AD mouse models. Additionally, chronic treatment with cTfRMAb alone or with cTfRMAb fusion proteins does not alter brain TfR expression, brain iron load, or plasma iron in mice [55,56], and it has a stable safety profile in primates [57] and humans [58]. 

## 4. Materials and Methods

### 4.1. cTfRMAb-EPO Fusion Protein

The cTfRMAb-EPO fusion protein was synthesized and formulated from ExpiCHO cells grown in serum-free ExpiCHO Expression Medium (Gibco, Gaithersburg, MD, USA) at a concentration of 0.78 mg/mL in 98 mM glycine, 148 mM NaCl, 28 mM Tris, and 0.01% Polysorbate 80 (pH = 5.5) by Genscript (Piscataway, NJ, USA) [14], and was sterile-filtered before use. The concentration of EPO was 0.16 mg/mL because the cTfRMAb-EPO is 20% EPO based on molecular weight [13]. The current study utilized cTfRMAb-EPO with the N292G mutation [14]. The cTfRMAb-EPO fusion protein was affinity-purified with a Protein G column and further fractionated by using Superdex 200 preparative-grade size exclusion chromatography (SEC). The final cTfRMAb-EPO fusion protein’s molecular weight and purity (92%) were confirmed using reducing and non-reducing SDS-PAGE and SEC HPLC. The produced cTfRMAb-EPO retained a high-affinity binding to the mouse TfR and EPO receptor with dissociation constants = 9.9 ± 2.1 ng/mL and 10.3 ± 0.61 ng/mL (~0.05 nM), respectively [14].

### 4.2. Mouse Treatment

Both male and female six-month-old hemizygous Tg (Prnp-MAPT*P301S) PS19Vle (PS19) mice (Jackson Laboratories, Bar Harbor, ME, USA) were utilized in the present study while following protocols approved by the University of California, Irvine, Institutional Animal Care and Use Committee. Six-month-old PS19 mice were selected for the current study because they show increased tau phosphorylation and gliosis (two key markers that were assessed in the current study) compared to younger PS19 mice. Further, because these mice have a median survival of ~9 months, the mice were treated up to 8 months of age (duration of treatment was 8 weeks) to limit the loss of mice due to premature death [21]. During the study, the mice were maintained under a 12-hour light–dark cycle and had constant access to food and water. For 8 weeks, IP injections were performed on the mice two or three days per week, alternatingly with cTfRMAb-EPO (PS19-cTfRMAb-EPO; 10 mg/kg, *n* = 10: female = 5, male = 5) (Supplemental Figure S1). The alternating injections of two or three doses per week were based on our previous work, wherein cTfRMAb-EPO was injected either two [15] or three [16] days a week. This cTfRMAb-EPO dose is equivalent to an EPO dose of 2 mg/kg because the cTfRMAb-EPO is 20% EPO based on molecular weight [15]. However, it should be noted that the plasma clearance of cTfRMAb-EPO is much faster than EPO [13,14], and hence, the systemic circulation and the resultant impact of 2 mg/kg EPO derived from the cTfRMAb-EPO is expected to be much lower than that of a 2 mg/kg dose of EPO alone. The IP route of administration results in higher plasma exposure of the cTfRMAb-EPO fusion protein with the N292G mutation, and therefore, it was used in the current study [22]. IP injections of an equivalent volume of saline were performed on the PS19 mice (PS19-Saline; *n* = 9: female = 5, male = 4) or age-matched wildtype (WT) littermates of PS19 mice (*n* = 12: female = 6, male = 6). The current study was run concurrently with a previous study published from our lab, and as a result, the control mice (PS19-Saline and WT) used in the current study belonged to the same cohort of mice used in our previous study [40]. Mouse body weights were recorded weekly for dosing. Adverse effects and abnormal behavior were monitored in all of the mice post-injection, as reported previously [55]. After 8 weeks of treatment and open-field testing (see below), a lethal dose of Euthasol (150 mg/kg, IP) was utilized to euthanize the mice. Terminal plasma was collected, and the mice were perfused transcardially with ice-cold phosphate-buffered saline (PBS). After perfusion, mice brains were collected, and the brain hemispheres were fixed in 4% paraformaldehyde (PFA) and cryoprotected in increasing sucrose concentrations (10%, 20%, and 30%) for sectioning and immunostaining [40]. 

### 4.3. Open-Field Behavioral Test

The mice were tested with the open-field behavioral test after 8 weeks of injections. Locomotion, hyperactivity, and anxiety were assessed with the open-field behavioral test as described previously [40,59]. The mice were acclimatized for at least 30 min before the test and were then placed in an open box (72 cm × 72 cm with 36 cm walls) with a center square (36 cm × 36 cm). The SMART Video Tracking Software (Panlab, Harvard Apparatus, Holliston, MA, USA) tracked the mice for five min. Locomotion hyperactivity was evaluated by measuring the mean speed, resting time, and total distance traveled by the mice. Anxiety was evaluated by measuring the amount of time the mice spent in the center. 

### 4.4. Immunostaining

Fixed and cryoprotected hemi-brains from nine to ten mice per group were sliced into 20 μm-thick sagittal sections at −25 °C using a cryostat (Micron Instruments, Simi Valley, CA, USA). The pTau immunostaining, for detecting pTau at Ser202 and Thr205 (AT8), and Iba1 immunostaining, for detecting microgliosis, utilized five tissue sections that were 600 µm apart per mouse as described previously [40]. Primary antibody omission on a subset of brain sections was used as the negative control to confirm primary antibody specificity for AT8 and Iba1. 

Briefly, tissue sections were washed in PBS and blocked with 0.5% bovine serum albumin (BSA) in PBS containing 0.3% Triton X-100 (TX100) for 60 min at room temperature (RT). For pTau staining, the tissue sections were incubated with 0.2% biotin-conjugated pTau (AT8 antibody, RRID:AB_223648, Thermo Fisher Scientific, Waltham, MA, USA) overnight at 4 °C. For Iba1 immunostaining, the tissue sections were incubated in 0.5 µg/mL anti-Iba1 rabbit antibody (RRID:AB_839504, Fujifilm Wako Chemical, Richmond, VA, USA) in PBS containing 0.3% TX100 and 0.5% BSA overnight at 4 °C. The pTau-stained sections were subsequently incubated with 0.5% Alexafluor 594 conjugated streptavidin (Biolegend, San Diego, CA, USA), and the Iba1-stained sections were incubated with 0.1% Alexafluor 488 donkey anti-rabbit IgG (RRID:AB_2563203, Biolegend, San Diego, CA, USA) in PBS containing 0.3% TX100 and 0.5% BSA in the dark for 2 h at RT. Stain-positive area (%) was quantified in the cerebral cortex, hippocampus, entorhinal cortex, and amygdala for each tissue section using NIH ImageJ Software (version 1.53e, Bethesda, MD, USA) with a threshold setting as described previously [40]. The overall stain-positive area was the average of the cerebral cortex, hippocampus, entorhinal cortex, and amygdala sections. Two independent observers who were blinded to the treatment groups analyzed the images.

### 4.5. Hematoxylin and Eosin (H&E) Staining

Hippocampal neuronal density was assessed in six to seven randomly selected mice per treatment group, based on sample size calculation, as previously described [40]. Three 20-µm sagittal mouse brain sections (~600 µm apart) were mounted onto glass slides and air-dried overnight. H&E staining was used to assess hippocampal neuronal loss as described before in PS19 mice [21] and in other models of neurodegeneration [60]. Briefly, H&E staining was performed with washes and incubation in acetal staining jars (Simport, Saint-Mathieu-de-Beloeil, QC, Canada) with water, Mayer’s Hematoxylin (Fisher Scientific, Waltham, MA, USA), Scott’s tap water/bluing reagent, Eosin Y (0.5% aqueous solution; Sigma Aldrich, St. Louis, MO, USA), ethanol, and xylene (Sigma Aldrich, St. Louis, MO, USA). The slides were cover-slipped with Permount mounting media (Fisher Scientific, Waltham, MA, USA). A light microscope (Motic, Richmond, BC, Canada) was utilized to image each slide, and different regions in the hippocampus (DG, CA1, CA2, and CA3) were imaged at 10x magnification. The areas occupied by the hippocampal neurons (µm^2^) in the granule cell layer of the DG and pyramidal layer of the CA1–3 regions were manually outlined, and all images were quantified with NIH ImageJ (version 1.53e, Bethesda, MD, USA). 

### 4.6. Statistical Analysis

Power analysis was performed using G*power 3.1.9.7, and based on an effect size ranging between 1.2–1.7 [16], a significance level of 5%, and a power of 80%, a sample size of 7–12 animals per group was calculated. For H&E analysis, based on an effect size ranging between 1.47–2.05 [40], a significance level of 5%, and a power of 80%, a sample size of 5–9 animals per group was calculated. All data are represented as mean ± SEM, and all statistical analyses were performed using GraphPad Prism 9 (GraphPad Software Inc., La Jolla, CA, USA). Due to similar treatment trends, the male and female data were combined. Outliers were identified using Grubb’s test, normality was determined using the Kolmogorov–Smirnov test, and equality of variance was confirmed using the Brown–Forsythe test. The open-field and histochemistry data were analyzed with a one-way ANOVA with Holm–Sidak’s multiple comparisons test. A two-way ANOVA with Holm–Sidak’s multiple comparisons test was performed on weekly weight measurements. A two-tailed *p* < 0.05 was considered statistically significant. 

## 5. Conclusions

Chronic dosing of the BBB-penetrating cTfRMAb-EPO fusion protein reversed altered anxiety and hyperactive phenotypes and reduced phosphorylated tau and microgliosis, demonstrating therapeutic effects in a PS19 transgenic mouse model of tauopathy. The results presented in this proof-of-concept study thereby offer promise for the use of this BBB-penetrating EPO molecule for tauopathies, including AD. 

## Data Availability

Data are contained within the article and Supplementary Materials.

## Supplementary Materials

The following supporting information can be downloaded at https://www.mdpi.com/article/10.3390/ph16040558/s1, Figure S1: (**A**) Schematic of the study design. (**B**) Weekly weights of the mice over the course of the 8-week treatment; Figure S2: (**A**) Total plasma tau in the cTfRMAb-EPO-treated mice following 8 weeks of treatment of six-month-old PS19 mice. (**B**) AT8-positive phospho-tau (Ser202, Thr205) in the PS19-Saline- and PS19-cTfRMAb-EPO-treated mice in whole-brain homogenates using Western blotting. (**C**) Correlation between the overall brain AT8-positive area and total plasma tau; Video S1: Open-field video for a representative WT mouse; Video S2: Open-field video for a representative PS19-Saline mouse; Video S3: Open-field video for a representative PS19-cTfRMAb-EPO mouse.
