# Supplementary material for: The Effects of a Blood–Brain Barrier Penetrating Erythropoietin in a Mouse Model of Tauopathy
Source: Pharmaceuticals (Basel). 2023 Apr 7;16(4):558. doi: 10.3390/ph16040558 (PMC10141171; doi:10.3390/ph16040558)
Supplement: Supplementary file 1 [file pharmaceuticals-16-00558-s001.zip › pharmaceuticals-2271282-supplementary.pdf]

Supplemental Methods

1. *Total human tau ELISA:*

Plasma human total tau was detected in diluted plasma (300-fold) using an ELISA as per the manufacturer's instructions (KHB0041, Thermo Fisher Scientific, MA, USA).

2. *Western Blot for phospho-tau (Ser202, Thr205):*

Frozen left hemi-brains were pulverized and homogenized in 2 volumes of RAB high salt buffer (G-Biosciences, MO, USA) (0.1 M MES, 1 mM EGTA, 0.5 mM $MgSO_4$, 0.75 M NaCl, 0.02 M NaF, 1 mM PMSF and 0.1% protease inhibitor mixture) and centrifuged at 20,800 x g for 40 min at 4 ˚C [1]. The supernatants were processed with 4× Laemmli buffer (Bio-Rad, CA, USA) with 10% beta-mercaptoethanol by boiling for 10 min. Protein samples (30-50 μg) were separated on 4-20% SDS-ready precast gels (Bio-Rad, CA, USA) and transferred to polyvinylidene fluoride membranes (Bio-Rad, CA, USA). Membranes were blocked using 5% non-fat milk in TBS for 1 h at room temperature and probed with the AT8 antibody (RRID:AB_223648, Thermo Fisher Scientific, MA, USA) overnight at 4 °C. Membranes were then probed with the high-sensitivity streptavidin-HRP (Thermo Fisher Scientific, MA, USA) at 1:2500 dilution in 3% non-fat milk for 1 h at room temperature followed by washing with TBST. ECL substrate reagent (Thermo Fisher Scientific, MA, USA) was used for detection and chemiluminescence was imaged using the Azure C500 gel imager (Azure Biosystems, CA, USA). Membranes were stripped and probed with an anti-β-actin antibody (1:1000 in 3% non-fat milk, RRID:AB_626632, Santa Cruz Biotechnology, TX, USA) as a loading control. Western blot bands were quantified using the NIH ImageJ software (version 1.53e, MD, USA), and data were normalized to PS19-Saline mice.

Supplemental Results

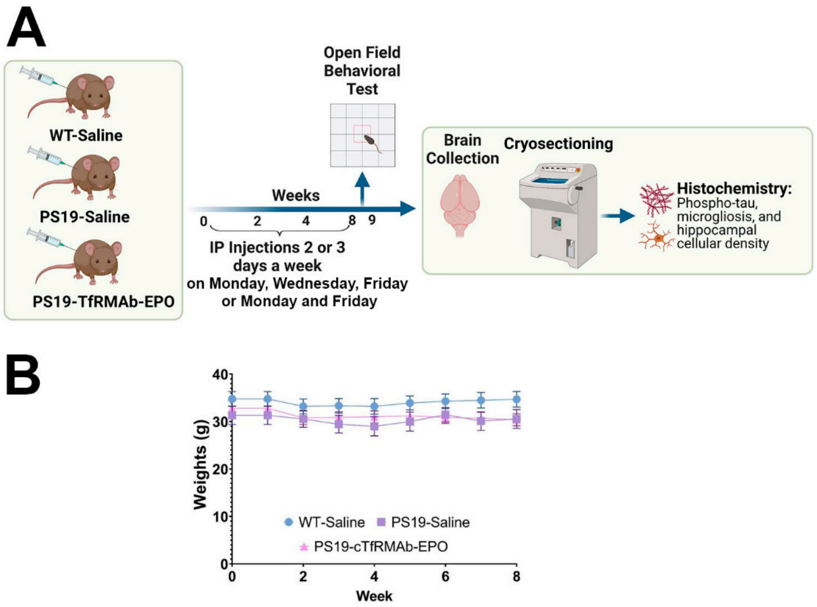

Figure S1: Schematic of the study design. Six-month-old male and female PS19 mice were treated with saline (PS19-Saline) or cTfRMAb-EPO (PS19-cTfRMAb-EPO; 10 mg/kg) IP two or three days per week alternatingly for 8 weeks. WT mice received an equivalent volume of saline following the same regimen. At the end of 8-weeks of treatment, open-field testing was performed, and brains were harvested for histology (**A**). Weekly weights of the mice over the course of the 8-week treatment. No significant difference was observed between the experimental groups and PS19-Saline treated mice throughout the study, and within each experimental group at baseline and following 8 weeks of treatment (**B**). Data are presented as mean ± SEM of n = 9-12 per treatment group. Two-way repeated measures ANOVA with Holm Sidak's post-hoc test was used to compare to the PS19-Saline mice.

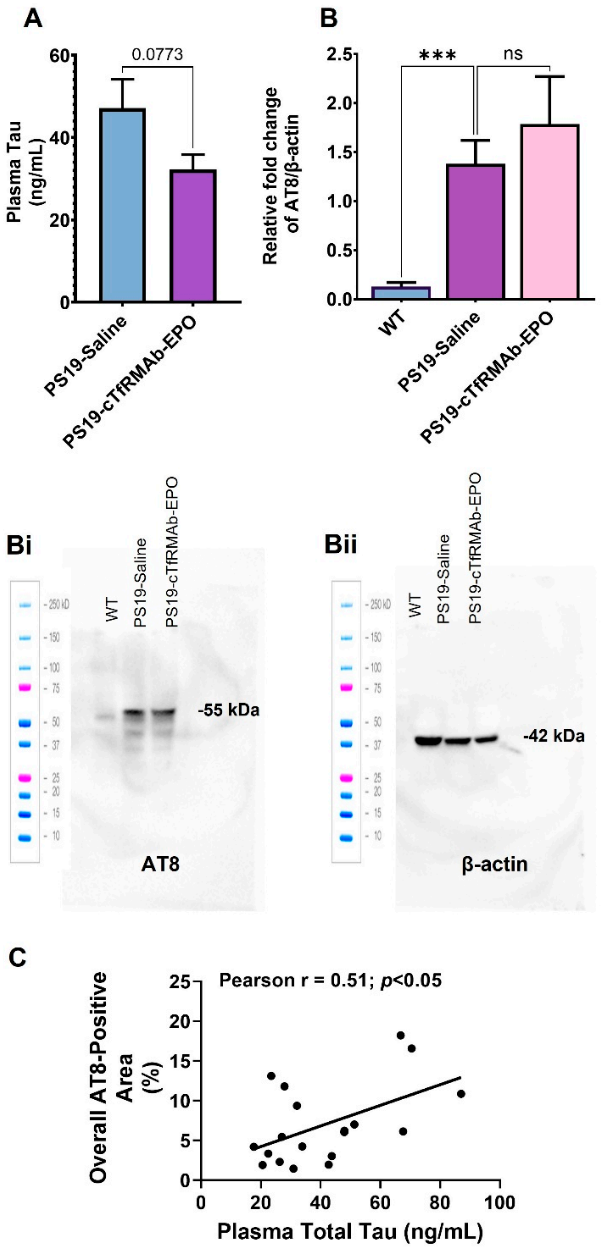

Figure S2: A trend towards a decrease in (**A**) total plasma tau in the cTfRMAb-EPO-treated mice following 8 weeks of treatment of six-month-old PS19 mice. (**B**) No significant difference in the AT8-positive phospho-tau (Ser202, Thr205) between the PS19-Saline- and PS19-cTfRMAb-EPO-treated mice in whole-brain homogenates using Western blotting. Representative full blot images showing the bands for **(Bi)** AT8 and **(Bii)** β-actin in the different experimental groups. Though we saw a marked reduction in the AT8 positive area in the cortex, hippocampus, amygdala, and entorhinal cortex using immunofluorescence, we did not observe a significant difference in the AT8

levels between saline-treated PS19 mice and cTfRMAb-EPO-treated PS19 mice using whole brain homogenates. Future studies using regional brain homogenates rather than whole-brain homogenates may address this discrepancy. A significant positive correlation between the overall AT8-positive area and total plasma tau **(C)**. Data are shown as mean ± SEM of n = 5-12 per group. ***p<0.001 compared to PS19-Saline. Student's t-test (**A**), one-way ANOVA with Welch correction for unequal variances followed by the Dunnett's T3 multiple comparisons test (**B**), and Pearson correlation r (**C**) were used.

References

1. Ou, W.; Yang, J.; Simanauskaite, J.; Choi, M.; Castellanos, D.M.; Chang, R.; Sun, J.; Jagadeesan, N.; Parfitt, K.D.; Cribbs, D.H.; et al. Biologic TNF-alpha inhibitors reduce microgliosis, neuronal loss, and tau phosphorylation in a transgenic mouse model of tauopathy. *J Neuroinflammation* **2021**, *18*, 312, doi:10.1186/s12974-021-02332-7.
